# Kurarinone Attenuates Collagen-Induced Arthritis in Mice by Inhibiting Th1/Th17 Cell Responses and Oxidative Stress

**DOI:** 10.3390/ijms22084002

**Published:** 2021-04-13

**Authors:** Kuo-Tung Tang, Chi-Chien Lin, Shih-Chao Lin, Jou-Hsuan Wang, Sen-Wei Tsai

**Affiliations:** 1 Program in Translational Medicine, National Chung Hsing University, Taichung 402, Taiwan; dirac1982@vghtc.gov.tw (K.-T.T.); lincc@email.nchu.edu.tw (C.-C.L.); 2 Faculty of Medicine, National Yang-Ming University, Taipei 112, Taiwan; 3 Division of Allergy, Immunology and Rheumatology, Taichung Veterans General Hospital, Taichung 407, Taiwan; 4 Institute of Biomedical Science, The iEGG and Animal Biotechnology Center, National Chung-Hsing University, Taichung 402, Taiwan; 5 Department of Medical Research, China Medical University Hospital, Taichung 404, Taiwan; 6 Department of Medical Research, Taichung Veterans General Hospital, Taichung 407, Taiwan; 7 Department of Pharmacology, College of Medicine, Kaohsiung Medical University, Kaohsiung 807, Taiwan; 8 Bachelor Degree Program in Marine Biotechnology, College of Life Sciences, National Taiwan Ocean University, Keelung 202, Taiwan; sclin@mail.ntou.edu.tw; 9 Department of Physical Medicine and Rehabilitation, Taichung Tzu Chi Hospital, Buddhist Tzu Chi Medical Foundation, Taichung 427, Taiwan; doris8569@smail.nchu.edu.tw; 10 School of Medicine, Tzu Chi University, Hualien 970, Taiwan

**Keywords:** kurarinone, arthritis, Th1, Th17, STAT1, STAT3, oxidative stress

## Abstract

Kurarinone is a flavanone, extracted from *Sophora flavescens* Aiton, with multiple biological effects. Here, we determine the therapeutic potential of kurarinone and elucidate the interplay between kurarinone and the autoimmune disease rheumatoid arthritis (RA). Arthritis was recapitulated by induction of bovine collagen II (CII) in DBA/1 mice as a collagen-induced arthritis (CIA) model. After the establishment of the CIA, kurarinone was given orally from day 21 to 42 (100 mg/kg/day) followed by determination of the severity based on a symptom scoring scale and with histopathology. Levels of cytokines, anti-CII antibodies, and the proliferation and lineages of T cells from the draining lymph nodes were measured using ELISA and flow cytometry, respectively. The expressional changes, including STAT1, STAT3, Nrf2, KEAP-1, and heme oxygenase-1 (HO-1) changes in the paw tissues, were evaluated by Western blot assay. Oxidative stress featured with malondiadehyde (MDA) and hydrogen peroxide (H_2_O_2_) activities in paw tissues were also evaluated. Results showed that kurarinone treatment reduced arthritis severity of CIA mice, as well as their levels of proinflammatory cytokines, TNF-α, IL-6, IFN-γ, and IL-17A, in the serum and paw tissues. T cell proliferation was also reduced by kurarinone even under the stimulation of CII and anti-CD3 antibody. In addition, kurarinone reduced STAT1 and STAT3 phosphorylation and the proportions of Th1 and Th17 cells in lymph nodes. Moreover, kurarinone suppressed the production of MDA and H_2_O_2._ All while promoting enzymatic activities of key antioxidant enzymes, SOD and GSH-Px. In the paw tissues, upregulation of Nrf-2 and HO-1, and downregulation of KEAP-1 were observed. Overall, kurarinone showed an anti-inflammatory effect by inhibiting Th1 and Th17 cell differentiation and an antioxidant effect exerted in part through activating the Nrf-2/KEAP-1 pathway. These beneficial effects in CIA mice contributed to the amelioration of their arthritis, indicating that kurarinone might be an adjunct treatment option for rheumatoid arthritis.

## 1. Introduction

Rheumatoid arthritis (RA) is a chronic autoimmune disease characterized by joint inflammation, cartilage damage, and joint destruction [1], that leads to negative impacts on the physical movement and life quality of patients. The pathogenesis of RA has been implied to be associated with uncontrolled autoimmunity and the resulting inflammation from antigen presenting cells, such as dendritic cells and macrophages, and lymphocytes, such as T and B cells [2,3,4]. The systemic inflammatory response targets the synovial membrane, leading to joint destruction through cartilage degradation and osteoclast activation [5,6]. Numerous studies have demonstrated the importance of activated T cells in RA pathogenesis. For example, experiments utilizing the collagen-induced arthritis (CIA) animal model have indicated the involvement of activated pro-inflammatory Th1 and Th17 cells and suppressed Treg cells could be involved in the RA pathogenesis [7]. Oxidative stress, a result of accumulated reactive oxygen species (ROS), also contributes to the synovitis development in RA. ROS can degrade proteoglycans and hypochlorous acid (HOCl) in the cartilage, leading to collagen fragmentations and proteoglycan synthesis suppression [8,9]. Increased oxidative stress has also been reported in RA patients [10]. 

At present, RA pharmacotherapy remains unsatisfactory, with various side effects [11,12,13]. Some RA patients are refractory to traditional disease modifying anti-rheumatic drugs (DMARDs) [14]. Some biological agents, though are good for refractory disease, cause serious infection problems [11]. There is a need for novel therapeutics with a favorable safety and efficacy profile. Kurarinone, a natural lavandulylated flavanone isolated from the medicinal herb *Sophora flavescens* Aiton, has immunosuppressive and antioxidant effects [15,16]. For example, it inhibits the differentiation of Th1/Th2/Th17 cells and promotes the differentiation of Treg cells. Its mechanism of action involves the regulation of multiple kinases signaling pathways, like JAK/STAT, T-cell receptor (TCR)-mediated Src family tyrosine kinase, PI3K/Akt, and p38 MAPK signaling. This results in suppression of psoriasis- and contact dermatitis-like inflammatory dermatitis in mice [16]. Kurarinone also inhibits the progression of murine autoimmune encephalomyelitis (EAE) by inhibiting Th1 and Th17 cell differentiation and proliferation [15]. Moreover, it activates the KEAP1/Nrf2 pathway, a major regulator of oxidative stress [17], and it overexpresses antioxidant enzymes, like heme oxygenase-1 (HO-1), thereby exerting immunosuppression on the lipopolysaccharide (LPS)-induced production of inflammatory mediators in RAW264.7 macrophages [18]. Based on these findings, we hypothesized that kurarinone has a therapeutic effect on RA through regulating Th1/Th17 cell balance and antioxidative activity. In this study, we attempted to evaluate kurarinone in the murine model of collagen-induced arthritis (CIA).

## 2. Results

### 2.1. Kurarinone Reduced the Severity of CIA 

**Figure 1:**
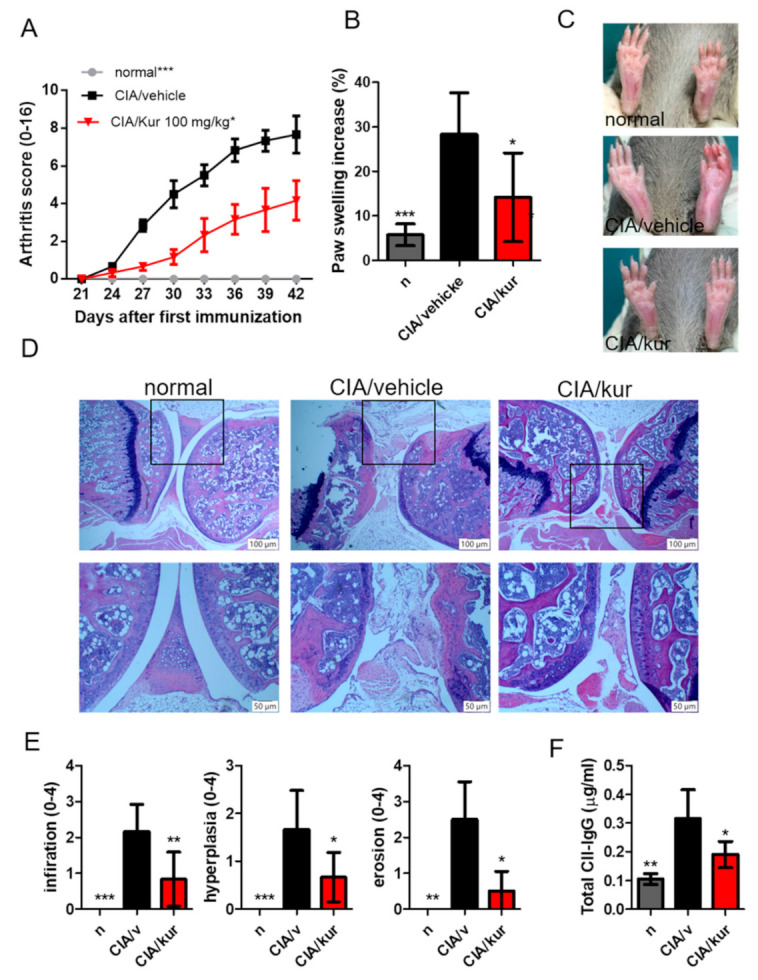
Effects of kurarinone on the severity of CIA. (**A**) Mice were immunized with bovine type II collagen in complete Freund’s adjuvant, and (**A**) clinical scores and (**B**) paw thickness was measured on day 42. (**C**) Representative images of a hind paw at day 42. (**D**) Mouse joints stained with hematoxylin and eosin (scale bar, 100 μM, 100×; 50 μM 200×). (**E**) Semiquantitative histological analysis. (**F**) Serum anti-CII IgG was analyzed by ELISA on day 42. Data are presented as mean ± SEM of 6 mice from one of three experiments. (*) *p* < 0.05, (**) *p* < 0.01, (***) *p* < 0.001 versus vehicle-treated CIA mice group (Two Way (**A**), or One Way (**B**,**E**,**F**) ANOVA followed by Tukey’s multiple comparison test).

To determine the effect on arthritis in CIA, symptom scores were measured [19]. As shown in Figure 1, the administration of bovine CII emulsion in DBA/1 mice led to the development of arthritic symptoms (Figure 1A), which included swelling and erythema of the hind paws (Figure 1B,C). Symptom scores of experimental mice were lower after daily treatment with 100 mg/kg kurarinone for three weeks. Consistent with that, histopathologic findings in these animals on day 42 after primary immunization showed less joint inflammation (synovitis), pannus formation, cartilage destruction, and bone erosions compared to the control (Figure 1D,E). Moreover, treatment with kurarinone also reduced serum levels of CII-specific IgG (Figure 1F).

### 2.2. Kurarinone Reduced Pro-Inflammatory Cytokine Levels in the Blood and Joints

**Figure 2:**
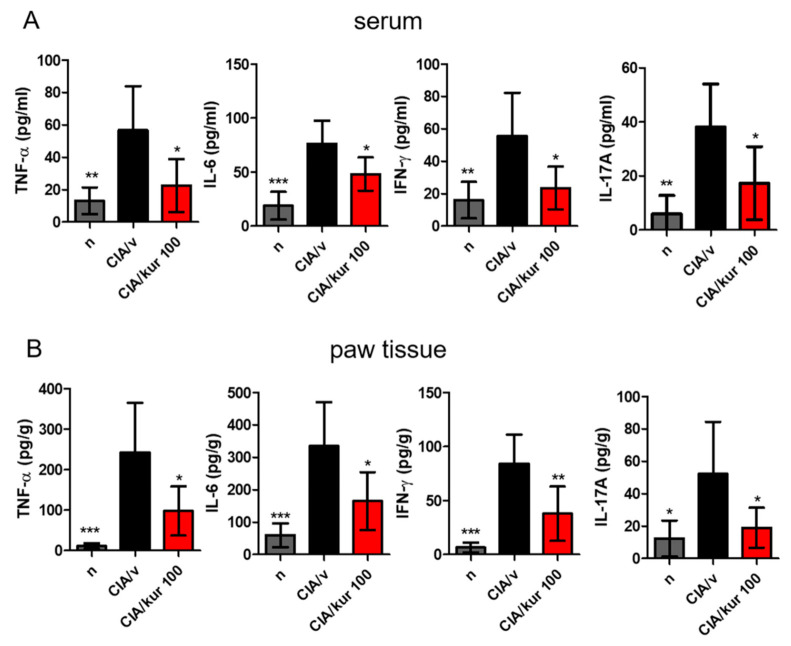
Effects of kurarinone on serum and paw proinflammatory cytokine productions in CIA mice. Hind paws and sera were collected on day 42 from mice. (**A**) Cytokines in serum and (**B**) paw homogenates were measured by ELISA assays. Data are presented as mean ± SEM of 6 mice from one of three experiments. (*) *p* < 0.05, (**) *p* < 0.01, (***) *p* < 0.001 versus vehicle-treated CIA mice group (One Way ANOVA followed by Tukey’s multiple comparison test).

Cytokine levels in serum and paw homogenates were measured using ELISA on day 42. As shown in Figure 2, kurarinone significantly reduced serum levels of TNF-α, IL-6, IFN-γ, and IL-17A when compared with those in vehicle-treated mice (Figure 2A). Similar results on pro-inflammatory cytokine levels were found in paw homogenates (Figure 2B). 

### 2.3. Kurarinone Treatment Attenuated CII-Specific T Cell Proliferation in CIA

**Figure 3:**
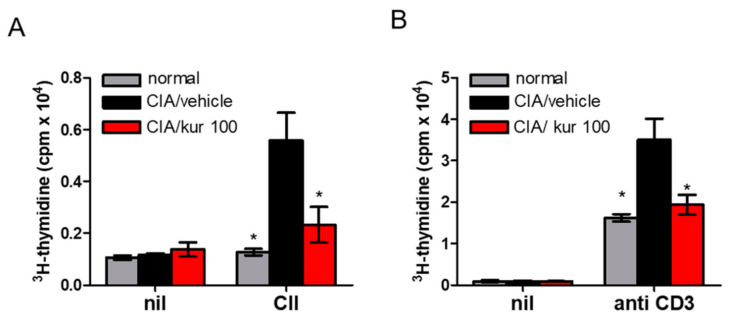
Effects of kurarinone on T cell proliferation in CIA. On day 42, cells from the inguinal LN were isolated for mice and were cultured for 96 hrs in the absence or presence of (**A**) 10 ug/mL CII or (**B**) anti-CD3 antibody. Proliferation was assessed after 96 h by [^3^H] thymidine incorporation in counts per minute (cpm). Data are presented as mean ±SEM of 6 mice from one of three experiments. (*) *p* < 0.05 versus vehicle-treated CIA mice group (One Way ANOVA followed by Tukey’s multiple comparison test).

Antigen-specific T-cell responses were examined on T cells isolated from the draining lymph nodes in CIA mice on day 42 after CII stimulation. While T cells obtained from vehicle-treated CIA mice showed a strong proliferative response to CII. Such a response was significantly suppressed in kurarinone-treated mice (Figure 3A). Kurarinone also significantly suppressed T cell proliferation upon stimulation with anti-CD3 antibody (Figure 3B). These results indicate that kurarinone had attenuated T cell proliferation after stimulation in CIA mice.

### 2.4. Kurarinone Altered Th1/Th17 Differentiation in Lymph Nodes of CIA Mice 

**Figure 4:**
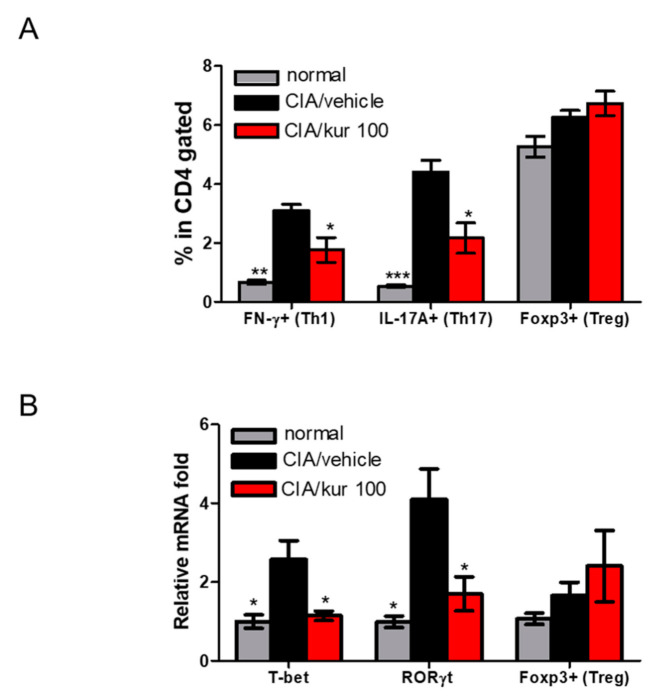
Effects of kurarinone on frequency of Th1 (CD4+ IFN-γ+ T cells), Th17 cells (CD4+ IL-17+ T cells) and Treg (CD4+Foxp3+ T cells) in the inguinal lymph nodes (ILNs). (**A**) Single cell suspensions were collected from ILNs on day 42, followed by stimulation with anti-CD3+ for 96 h and then stained with anti-CD4, anti-IFN-γ, anti-IL-17A, or anti-Foxp3 Abs and analyzed by flow cytometry. Statistical analysis of the percentages of Th1, Th1, and Treg in the LN. (**B**) The mRNA relative expressions of specific transcription factors for different CD4+ T cells subsets were calculated by quantitative RT-PCR using the ΔΔ CT method with the normal control group as calibrator. Data are presented as mean ± SEM of 6 mice from one of three experiments. (*) *p* < 0.05, (**) *p* < 0.01, (***) *p* < 0.001 versus vehicle-treated CIA mice group (One Way ANOVA followed by Tukey’s multiple comparison test).

We further explored the possible mechanisms involved in the therapeutic effects of kurarinone on CIA mice. The abundance of Th1, Th17, and Treg in the inguinal lymph nodes was analyzed using flow cytometry. As illustrated in Figure 4A, the percentages of CD4^+^IFN-γ^+^ Th1 cells and CD4^+^IL-17^+^ Th17 cells isolated from the LN of kurarinone-treated CIA mice was lower than those of vehicle-treated group. However, CD4^+^Foxp3^+^ Treg cells appeared to have increased after kurarinone, but without reaching statistically significant levels (Figure 4A and Supplementary Figure S1). We also analyzed the lymph nodes using qPCR for the expressions of transcription factors, such as T-bet, RORγt, and Foxp3. Lower expressions of T-bet and RORγt in the lymph nodes were found in kurarinone-treated CIA mice, whereas the expression level of Foxp3 was unchanged (Figure 4B). Altogether, these findings suggest that the suppression of Th1 and Th17 differentiation is involved in the mitigation of arthritic symptoms in kurarinone-treated CIA mice. 

### 2.5. Kurarinone Suppressed STAT1 and STAT3 Phosphorylations in the LN of Kurarinone-Treated Mice 

**Figure 5:**
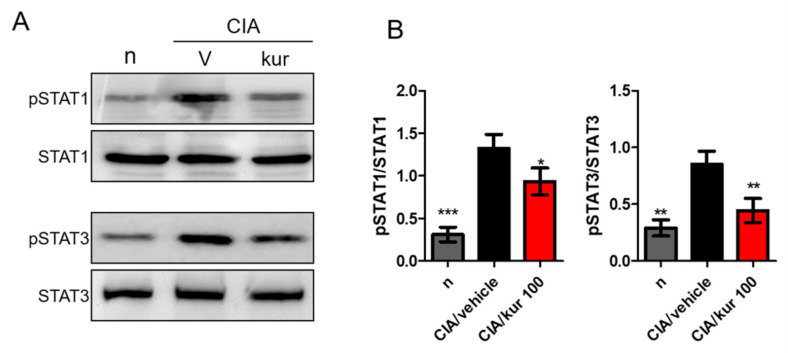
Effects of kurarinone on the phosphorylation of p-STAT1 and p-STAT3 in lymph nodes. Single cell suspensions were collected from ILNs on day 42, the protein expression levels of p-STAT1, STAT1, p-STAT3, and STAT3 were measured using Western blots. (**A**) Representative images of Western blot and (**B**) Densitometric analysis for protein expressions was performed using ImageJ software. Data are presented as mean ± SEM of 6 mice from one of three experiments. (*) *p* < 0.05, (**) *p* < 0.01, (***) *p* < 0.001 versus vehicle-treated CIA mice group (One Way ANOVA followed by Tukey’s multiple comparison test).

STAT1 and STAT3 are crucial transcription factors for Th1 and Th17 differentiation, respectively. We, therefore, measured expression levels of p-STAT1, STAT1, p-STAT3, and STAT3 in drained lymph nodes using Western blotting. Notably, significantly higher expression of phosphorylated STAT1 and STAT3 was found in the lymph nodes of CIA mice, and kurarinone treatment significantly suppressed these phosphorylation events (Figure 5A,B).

### 2.6. Kurarinone Suppressed Joint Oxidative Stress and Increased the Activity of Antioxidant Enzymes in CIA Mice

**Figure 6:**
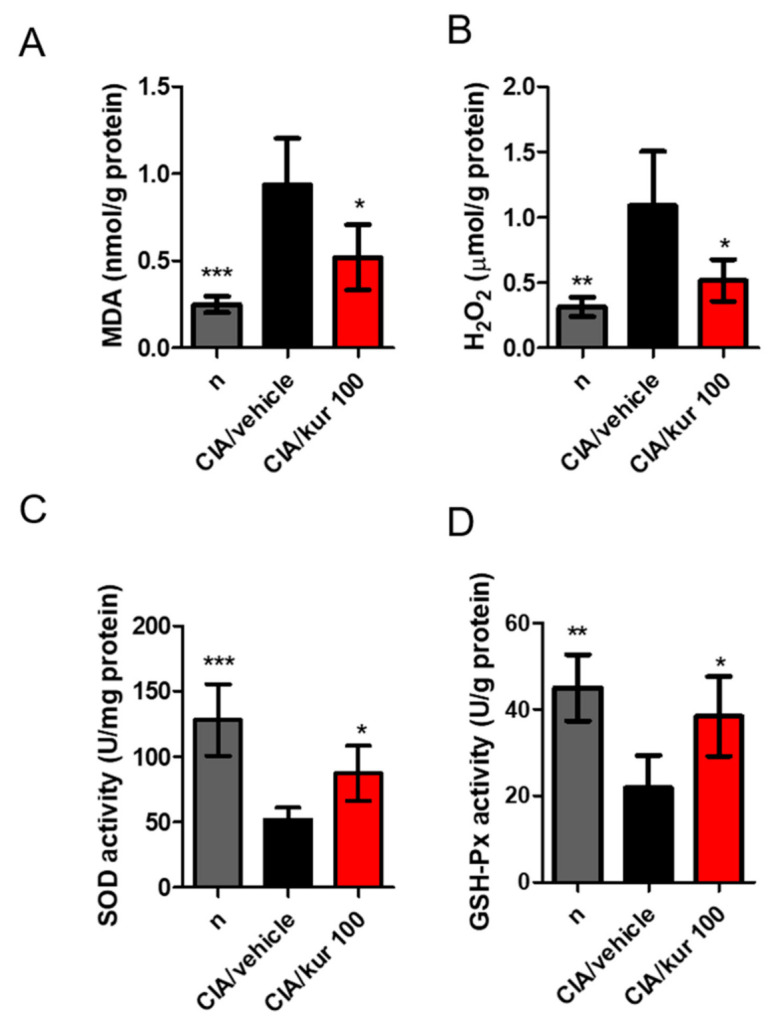
Effects of kurarinone on MDA, H_2_O_2_, SOD, and GSH-Px activities in paw tissues of CIA mice. On day 42, mice were sacrificed and the (**A**) MDA, (**B**) H_2_O_2_; (**C**) SOD, and (**D**) GSH-Px, expressions or activities in the paw tissues were measured as described in the methods and materials section. Data are presented as mean ± SEM of 6 mice from one of three experiments. (*) *p* < 0.05, (**) *p* < 0.01, (***) *p* < 0.001 versus vehicle-treated CIA mice group (One Way ANOVA followed by Tukey’s multiple comparison test).

It has been suggested that the release of free radicals and oxidizing agents, such as malondialdehyde (MDA) and hydrogen peroxide (H_2_O_2_), contributes to disease severity in RA. Therefore, we examined the effect of kurarinone treatment on oxidative stress in the joints of CIA mice. On day 42, MDA and H_2_O_2_ concentrations were drastically increased in the joints of vehicle-treated CIA mice, when compared with the control mice. This increase was significantly reversed by kurarinone treatment, as shown in Figure 6A,B. In line with this, the activity of antioxidant enzymes, like SOD and GSH-Px in paw tissues, significantly dropped in vehicle-treated CIA mice compared with control mice. Kurarinone treatment significantly increased the activity of these antioxidants when compared with vehicle-treated CIA mice (Figure 6C,D). 

### 2.7. Kurarinone Activated the Nrf2 Pathway in Paw Tissues of CIA Mice

**Figure 7:**
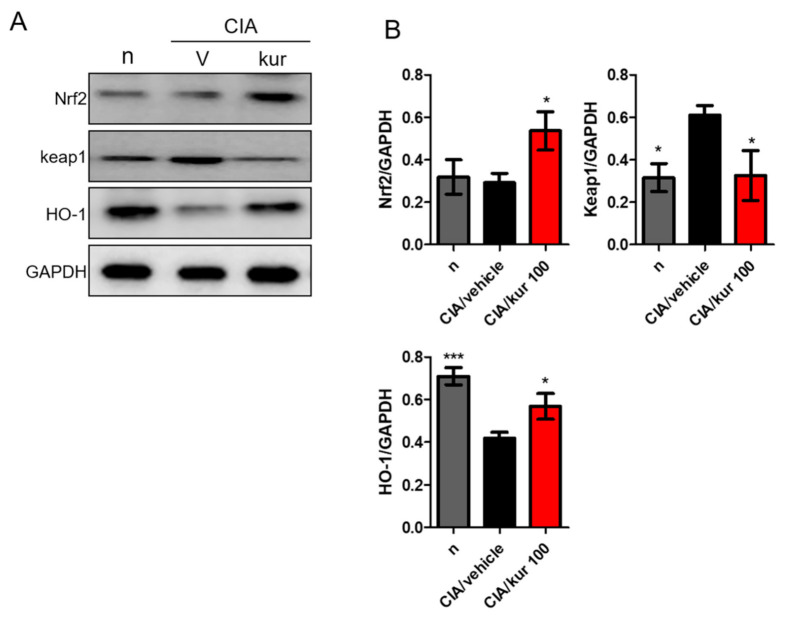
Effects of kurarinone on the expression of NRF2 pathway hind paw homogenates. Hind paws were collected on day 42, the protein expression levels of NRF2, Keap1, and HO-1 were measured using Western blots. (**A**) Representative images of Western blot and (**B**) Densitometric analysis for protein expressions was performed using ImageJ software. Data are presented as mean ± SEM of 6 mice from one of three experiments. (*) *p* < 0.05, (***) *p* < 0.001 versus vehicle-treated CIA mice group (One Way ANOVA followed by Tukey’s multiple comparison test).

Nuclear factor, erythroid 2-like 2 (Nrf-2) is a transcription factor that plays a major role in cell protection against oxidative stress. It induces antioxidant enzymes that inactivate reactive oxygen species, like heme oxygenase-1 (HO-1), GPX, and CAT. Kurarinone is known to activate the KEAP1/Nrf2/HO-1 pathway to exert immunomodulatory effects [18]. Therefore, we further studied kurarinone’s effects on expressions of Nrf2 and KEAP1 in the paw tissues of mice. We found that Nrf2 and HO-1 expressions were significantly higher in paw tissues of kuranionine-treated CIA mice (Figure 7A,B), whereas the KEAP1 expression was down-regulated. These findings suggest that Nrf2 might play an important role in the therapeutic effects of kuraninone in CIA mice. 

## 3. Discussion

Our present study is the first to demonstrate that kuraninone is therapeutically effective against CIA in a mouse animal model. The beneficial effects of kuraninone are evident by the amelioration of arthritic symptoms and histological improvements. The results are likely related to anti-inflammatory and antioxidant properties of kuraninone. The underlying mechanism includes the suppression of B and T cell responses and the elimination of oxidative stress through the up-regulation of Nrf2. Taken together, kuraninone is a potentially beneficial treatment for RA through a variety of mechanisms. 

Kuraninone is a natural flavonone purified from Sophora *flavescens* Aiton, one of the traditional Chinese medicinal herbs. *Sophora flavescens* is known to have anti-inflammatory effects [20] and is used to treat inflammatory diseases such as ulcerative colitis [21], Behcet syndrome [22], arthritis [23], pulmonary fibrosis [24], asthma [25], and influenza infection [24]. Other studies have also reported anti-inflammatory effects of kuraninone through its potent inhibition of cyclooxygenase (COX)-1 [26]. Kurarinone also attenuates levels of inducible nitric oxide synthase-dependent NO, interleukin (IL)-1β. It also activates extracellular signal-regulated kinase (ERK)1/2, c-Jun N-terminal kinase (JNK), p38 MAP kinases, and nuclear factor kappa-light-chain-enhancer of activated B cells (NF-kB) in lipopolysaccharide (LPS)-stimulated RAW264.7 macrophages [18,27]. Key inflammatory cytokines (e.g., IFN-γ, IL-1α, IL-1β, IL-4, IL-6, IL-17A, IL-22, and TNF-α) and chemokines (e.g., CCL17, CCL20, CCL27, and CXCL1) are reduced by kurarinone in mice with autoimmune encephalomyelitis (EAE). Moreover, kuraninone inhibits Th1, Th2, and Th17 cell differentiation and proliferation, both in vitro and in vivo. These effects are likely related to its inhibition of EAE progression [15] and chronic inflammatory skin diseases such as psoriasis and contact dermatitis [16]. This study’s results align with other reports readily available in the literature. Specifically, kuraninone exerted anti-inflammatory effects in CIA mice suppressed production of pro-inflammatory cytokines and anti-CII antibody, T cell activation, and lowered the proportions of Th1 and TH17 cells. 

In addition, kuraninone is reported to have antioxidant properties in assays with 2,2′-azino-bis-3-ethylbenzothiazoline-6-sulfonic acid (ABTS), peroxynitrite (ONOO^–^), and total ROS [28], and it inhibits oxidation of low-density lipoprotein [29]. Furthermore, treatment with kuraninone in cultured cells protects them against tert-butylhydroperoxide (t-BHP) and TNF-α/IFN-γ-induced intracellular ROS generation [16,28]. In terms of cytoprotection, KEAP1-Nrf2 pathway is the major regulatory mechanism of endogenous and exogenous oxidative stress [17]. To be noted, the treatment of prostate cancer PC3 cells with kuraninone downregulates the expression of KEAP1 and subsequently activates Nrf2, which increases the expression of heme oxygenase-1 (HO-1), an antioxidant enzyme [18]. However, this effect is not found in mouse hippocampal HT22 cells treated with kuraninone [30]. This study results in kuranionine-treated CIA mice having an upregulation of Nrf2 and HO-1, and downregulation of KEAP-1. Taken together, the antioxidative ability of kuraninone is evident. When examining the contribution of oxidative stress to the inflammatory response [31,32], these properties further enhance the anti-inflammatory effects of kuraninone. 

A number of animal studies demonstrate the importance of pathogenic Th1 and Th17 cells in RA [33,34,35]. In addition, human studies reported similar findings early in disease progression. Higher levels of Th1 cells and IFN-γ are present in the synovial fluid and tissues in RA patients [36,37,38]. Other researchers also reported increased proportions of Th17 cells in the blood and bone marrow [39,40], and higher levels of IL-17 in the blood and joint fluid of RA patients [41,42,43]. Additionally, these levels correlate with disease progression. Based on these studies, abnormally upregulated Th1 and Th17 responses contribute to the generation of RA. Although mechanisms underlying the beneficial effects of kuraninone in CIA remain elusive, here we speculate that the therapeutic efficacy of kuraninone in CIA is partly due to the suppression of Th1 and Th17 responses. We further showed that kuraninone inhibited Th1 and Th17 responses in CIA mice via the suppression of STAT1 and STAT3 signaling, which in turn reduces T-bet and RORγt expression. Our results are compatible with reports on mouse CD4+ T cells, and human keratinocytes, and peripheral blood mononuclear cells, in which STAT1 and STAT3 activation is suppressed after kuraninone treatment [16]. 

In regards of T cell differentiation by kurarinone, macrophage is an antigen presenting cell that inevitably needs to be taken into consideration. While we did not provide further evidences related to macrophages, previous studies may have revealed several clues to illustrate the role of macrophages in regulating T cell functions under kurarinone treatment. It was shown that LPS activated murine macrophages, Raw 264.7 cells, to induce iNOS-dependent NO production and ROS generation as well as inflammatory cytokines, such as TNF-α, whereas kurarinone inhibited such activation via suppressing NFκB and mitogen-activated protein kinase pathways, subsequently inhibiting NO and ROS [27]. More recently, Nishikawa et al. also utilized LPS to stimulate RAW 264.7 cells and treated with kurarinone. They further confirmed that kurarione induced antioxidant enzymes, such as HO-1, to suppress iNOS and IL-1β production, rendering kurarinone being a potent antioxidant [18]. Kurarinone may also directly induce naïve T cell differentiation without the involvement of macrophages. Xie et al. purified naïve T cells and treated with kurarinone where they found that both Th1 and Th2 differentiation of T cells were dose-dependently inhibited by kurarinone [15]. Kurarinone was also shown to regulate JAK/STAT pathway to reduce the CD4 T cell differentiation [16]. Taken together, it would be reasonable to assume that kurarinone could likely inhibit T cell functions directly and indirectly through influencing macrophages.

Despite not being addressed in our study, previous studies have revealed the possible mechanisms in bone remodeling involved in RA pathogenesis. Wang et al. found that kurarinone could facilitate the differentiation of osteoblastic cells UMR 106 cells, promoting bone formation [44]. Also, a flavonoid, (2S)-2’-methoxykurarinone, which is highly similar to kurarinone in chemical structure and also abundant in *Sophora flavescent* roots, was shown by Kim et al. to downregulate the expression of receptor activator of nuclear factor-κB ligand (RANKL), subsequently inhibiting the RNAKL-induced osteoclast differentiation [45]. As a result, it is potential that kurarinone could influence the hemostasis of osteoclastogenesis and osteoblastogenesis in RA.

Kurarinone dosage used in this study showed no toxicity in mice based on body weight, behavior, and appearance [46]. Nevertheless, kuraninone has been reported to be hepatotoxic in rats [47,48]. Kurarinone could also accumulate in the liver and inhibit fatty acid β-oxidation, resulting in lipid accumulation and liver injury, a condition akin to hepatic steatosis [48]. While we have demonstrated that kurarinone can potentially ameliorate arthritic symptoms in this study, its toxicity should be thoroughly investigated before proceeding to any human studies.

## 4. Material and Methods

### 4.1. Animal Experiments

Female DBA/1J mice (20–22 g in weight) of eight-week-old were used. DBA/J strain mice were purchased from Jackson Laboratory (Bar Harbor, MA, USA) and were maintained under specific-pathogen-free (SPF) conditions with food and water ad libitum. All animals were treated in accordance with the Institutional Animal Care and Use Committee (IACUC) of National Chung Hsing University (NCHU), and the experiment was approved by the Committee on Animal Research and Care in NCHU (NO. 110030). 

### 4.2. Establishment of CIA Animal Model and Experimental Grouping

The mouse model of collagen type II-induced arthritis (CIA) was used as described previously [49]. Briefly, chicken type II collagen (Chondrex, Inc., Woodinville, WA, USA) 2 mg/mL (dissolved in 10 mM acetic acid solution) was mixed with an equal volume of complete Freund’s adjuvant (CFA) (Sigma-Aldrich, St. Louis, MO, USA) (containing *Mycobacterium tuberculosis* H37RA) and then emulsified via repeated aspiration in sterile tubes to form an emulsion of type II collagen. The mixture (100 μL/mouse) was intradermally injected on day 0 in the tail approximately 1.5 cm distal to the base. Twenty-one days later, an immunization second booster was given, using the same concentration of collagen II but this time prepared in incomplete Freund’s adjuvant (IFA) (Sigma-Aldrich, MO, USA). Kurarinone was purchased from Sigma Aldrich Co. (MO, USA) and dissolved in the corn oil (Sigma-Aldrich, MO, USA)/DMSO (Santa Cruz Biotechnology Inc., Dallas, TX, USA) vehicle (*v*/*v*, 95/5). After 1-week adaptive breeding, all DBA1/J mice were randomly divided into three groups of equal numbers (*n* = 6) as: (1) normal/control group, in which each animal was administered a 100 μL of corn oil/DMSO from day 21 to day 41; (2) the CIA + vehicle group; and (3) the CIA + 100 mg/kg kurarinone group. We also administered 25 and 50 mg/kg kurarinone in CIA mice but no therapeutic effect was found (Supplementary Figure S2). The mice in groups 2 and 3 were then given either corn oil/DMSO or kurarinone via oral gavage (100 μL) every day from day 21 to day 41. 

### 4.3. Mouse Arthritis Scoring System

The two investigators are specialists in these types of animal models and were blind to the experimental groupings from day 21 post-immunization. Arthritis assessment included items like erythema, edema, joint rigidity, and swelling levels of paws as measured with micro-calipers. Paw volumes were measured using a plethysmometer 37,140 (Ugo Basile SRL, Comerio, VA, Italy). The scoring of arthritis grades was based on a scale from 0 to 4, on all four paws of each animal as follows: 0: no swelling and redness; 1: the presence of redness/and or swelling of one joint or one digit; 2: the involvement of two joints; 3: more than two joints involved; 4: severe redness and swelling of the whole paw and all digits. The maximal arthritis score for each paw is 4, and therefore, the maximal total score is 16 for each animal.

### 4.4. Histological Analysis

Mice were sacrificed on day 42. Their knee joints were sectioned in the sagittal plane. Embedding knee joints into paraffin blocks to support the tissue structure. Blocks were cut into 5 μM thick sections, mounted onto microscope slides for analysis, and stained with hematoxylin and eosin (H&E). Histopathological changes of the joints and scoring of histopathological damages were determined under light microscopy, according to the previously defined parameters [50]. H&E sections were thus analyzed regarding the degree of cell infiltration, synovial hyperplasia, and cartilage destruction. The graded score for each feature ranged from 0 to 4 (0, no change; 1, mild change; 2, moderate change; 3, severe change; 4, total destruction of joint architecture). The maximum score for each knee was 8.

### 4.5. Measurement of Pro-Inflammatory Cytokine Levels

Paw tissues and serum from DBA/1 mice were collected by the end of day 42. Hind paws were homogenized in ice-cold saline using a tissue homogenizer (Mini-BeadBeater-1, BioSpec, Bartlesville, OK, USA) at 4800 rpm for 3 min. After centrifuging at 3000 rpm (4 °C, 10 min, twice), hind paw homogenates were harvested. Blood was withdrawn from the submandibular vein and centrifuged at 3000 rpm (4 °C, 10 min, twice) to collect serum. Levels of TNF-α, IL-6, IFN-γ, and IL-17A were measured in both serum and paw homogenates using murine ELISA kits (Biolegend, San Diego, CA, USA) according to the manufacturer’s instructions.

### 4.6. Draining Lymph Node (dLN) Cells Proliferation Assay

DBA/1 mice were killed on day 42, and inguinal dLN cells (4 × 10^5^ cells/well) were cultured with chicken CII (50 μg/mL) at 37 °C for 96 h, or with concanavalin A (ConA) (5 µg/mL) at 37 °C for 48 h. During the last 18 h of the incubation, cells were pulsed labeled with [^3^H]-thymidine (1 μL Ci/well; NEN-DuPont, Boston, MA, USA) overnight, and radioactivity incorporation was assessed by liquid scintillation counting (Beckman Instruments, Palo Alto, CA, USA). 

### 4.7. Fluorescence-Activated Cell Sorting (FACS)

Cell suspensions were prepared from lymph nodes obtained in sacrificed mice after 42 days. Cells (3 × 10^5^) were seeded in a 60-mm dish and cultured with Con A (5 µg/mL) for 24 h. During the last 4 h of culture, 10 μg/mL brefeldin A was added. Afterwards, cells were trypsinized and washed in phosphate buffered saline (PBS), and cells so collected were stained with phycoerythrin- (PE-) anti-CD4 antibody for 45 min (BD Biosciences, San Diego, CA, USA), washed twice with 0.1 BSA in PBS. Cells were then permeabilized and fixed with cytofix/Cytoperm Plus solution (#51-2090KZ, BD Biosciences, San Diego, CA, USA) for 20 min. Finally, cells were stained with FITC-anti-IL-17A (#506907, Biolegend, San Diego, CA, USA), FITC-anti-IFN-γ (# RM9001, eBioscience, Waltham, MA, USA), and with anti-Foxp3 (#560407, eBioscience, Waltham, MA, USA). Cells were analyzed with the AccuriTM C5 cytometer (Accuri Cytometers, Ann Arbor, MI, USA) and data processed with the BD Accuri™ C6 Plus software.

### 4.8. Quantitative Real-Time (RT)-PCR Analysis

Cell suspensions were First prepared from lymph nodes of mice sacrificed after 42 days. Total RNA was extracted using Trizol reagent, and 2 µg of the RNA was RT reacted with an oligo-dT primer. Real-time PCR was carried out using the ABI 7500 Fast real-time detection system (Applied Biosystems; Thermo Fisher Scientific, Inc., Bangalore, IN, USA) with Fast SYBR™ Green Master Mix (Cat. No. 4385618, Applied Biosystems; Thermo Fisher Scientific, Woolston Warrington, UK). Cycling conditions were 95 °C for 10 min, followed by 40 cycles of 95 °C for 10 s, 60 °C for 30 s, and 72 °C for 30 s. For quantification, the target gene level was normalized to the internal standard gene, GAPDH. Primers used in the study are listed as below. RORγt forward, 5′-CCGCTGAGAGGGCTTCAC-3′, and reverse, 5’-TGCAGGAGTAGGCCA CATTACA-3’ [1]; T-bet, forward, 5’-GATCATCACCAAGCAGGGACG-3’, and reverse, 5’-TCCACACTGCACCCACTTGC-3’ [2]; Foxp3, 5’- CAAGTTCCACAACATGCGAC -3’ and reverse, 5’- ATTGAGTGTCCGCTGCTTCT-3’ [3]; Glyceraldehyde 3-phosphate dehydrogenase (GAPDH), forward, 5’–CGTGTTCCTACCCCCAATGT−3’and reverse, 5’ TGTCATCATACTTGGCAGGTTTCT−3′. All data were normalized against the expression level of GAPDH. The relative expression levels of genes in the experimental group were calculated with the ΔΔ CT method, and a water-treated control group acted as the calibrator.

### 4.9. Western Blot Analysis 

Cells isolated from inguinal lymph nodes (ILNs) on day 42 were stimulated with Con A (5 µg/mL) (Sigma-Aldrich, St. Louis, MO, USA) for 6 h. These cells were harvested and cell pellets were lysed on ice in a RIPA buffer containing 1% protease inhibitor cocktail (Sigma-Aldrich, St. Louis, MO, USA). In addition, the hind paw tissues were homogenized in a 100 μL tissue RIPA lysis buffer for 30 min. Total protein concentration was measured with the bicinchoninic acid (BCA) assay kit (Thermo Fisher Scientific, Waltham, MA, USA). Normalized amounts of protein were separated on 10% SDS-PAGE at 100 V for 1.5 h, before transferring onto the polyvinylidene difluoride (PVDF) membrane (Millipore, Billerica, MA, USA) at 300 mA for 1 h. The membrane was blocked with BlockPRO™ Protein-Free Blocking Buffer for 1.5 h at room temperature and then incubated at 4 °C overnight with the following: anti-pSTAT1 (Cat# AF2894, R & D systems, Minneapolis, MN, USA), anti-STAT1 (Cat# 9172, Cell Signaling Technology, Danvers, MA, USA), anti-pSTAT3 (Cat# MAB1799, R & D systems, Minneapolis, MN, USA), anti-STAT3 (Cat# ab119352), anti-Nrf2 (Cat# ab89443), anti-keap1 (Cat# ab119403), anti-HO-1 (Cat# 223349) and glyceraldehyde 3-phosphate dehydrogenase (GAPDH) (Cat# ab8245, Abcam, Cambridge, MA, USA). Subsequently, the membrane was incubated with the appropriate secondary antibody, and immunoreactive bands were visualized using the LumiFlash™ Ultima Chemiluminescent substrate, HRP system (Visual protein, Taipei City, Taiwan; LF08–500). Each membrane was re-probed with the antibody against GAPDH as the internal control for an equal protein loading. The band density was analyzed with the ImageJ v1.47 program (Bethesda, Rockville, MD, USA). 

### 4.10. Measurement of the Concentrations of Oxidative Markers

On day 42, the hind paw tissues were homogenized with 100 μL ice-cold tissue RIPA lysis buffer. The homogenate was incubated at 4 °C for 30 min and centrifuged at 12,000× *g* at 4 °C for 20 min. The supernatant was obtained as the total protein extract. Total protein concentration was measured with the bicinchoninic acid (BCA) assay kit. 

The level of malondialdehyde (MDA) was determined with the Lipid Peroxidation (MDA) Assay Kit (Colorimetric/Fluorometric) (Cat# ab118970, Abcam, Cambridge, MA, USA) according to the manufacturer’s instructions. Superoxide Dismutase (SOD) and glutathione peroxidase (GSH-Px) activities were determined with the Superoxide Dismutase (SOD) Colorimetric Activity Kit (Cat# EIASODC, Thermo Fisher Scientific, Waltham, MA, USA) and glutathione peroxidase ELISA Kit (Cat#11352, Cusabio Biotech Co., Ltd., Wuhan, China), all according to manufacturer’s instructions. The amount of hydrogen peroxide (H_2_O_2_) was estimated using the Hydrogen Peroxide Assay Kit (ab102500, Abcam, Cambridge, MA, USA) according to the manufacturer’s instructions. 

### 4.11. Statistical Analysis

Numerical data were presented as mean ± SD. Comparisons among multiple treatments were Tukey’s HSD (honest significant difference) test after one-way ANOVA or two-way ANOVA using GraphPad Prism (version 8 for Windows; GraphPad Software, La Jolla, CA, USA). Statistical significance was set at *p* < 0.05.

## 5. Conclusions

In conclusion, our results showed that kuraninone was a beneficial treatment against CIA development. Kuraninone exerted its therapeutic effects through the suppression of inflammatory cytokines and the modulation of B and T cell immune responses. Moreover, kuraninone’s antioxidant properties further alleviated inflammation. Add-on kuraninone treatment could therefore be an alternative when treating RA patients. However, its use in conjunction with current RA therapies requires further studies.

## Data Availability

The data that support the findings of this study are available from the corresponding author upon reasonable request.

## Supplementary Materials

The following are available online at https://www.mdpi.com/article/10.3390/ijms22084002/s1, Figure S1: Frequencies of IFN-γ+CD4+ (Th1), IL-17A+CD4+ (Th17) and Foxp3+CD4+ (Treg) in the inguinal lymph nodes (ILNs), Figure S2: Effects of kurarinone (25 and 50 mg/kg) on the severity of CIA.
