# Supplementary figures and images for: Kurarinone Attenuates Collagen-Induced Arthritis in Mice by Inhibiting Th1/Th17 Cell Responses and Oxidative Stress
Source: Int J Mol Sci. 2021 Apr 13;22(8):4002. doi: 10.3390/ijms22084002 (PMC8069507; doi:10.3390/ijms22084002)

Supplemental Fig S1

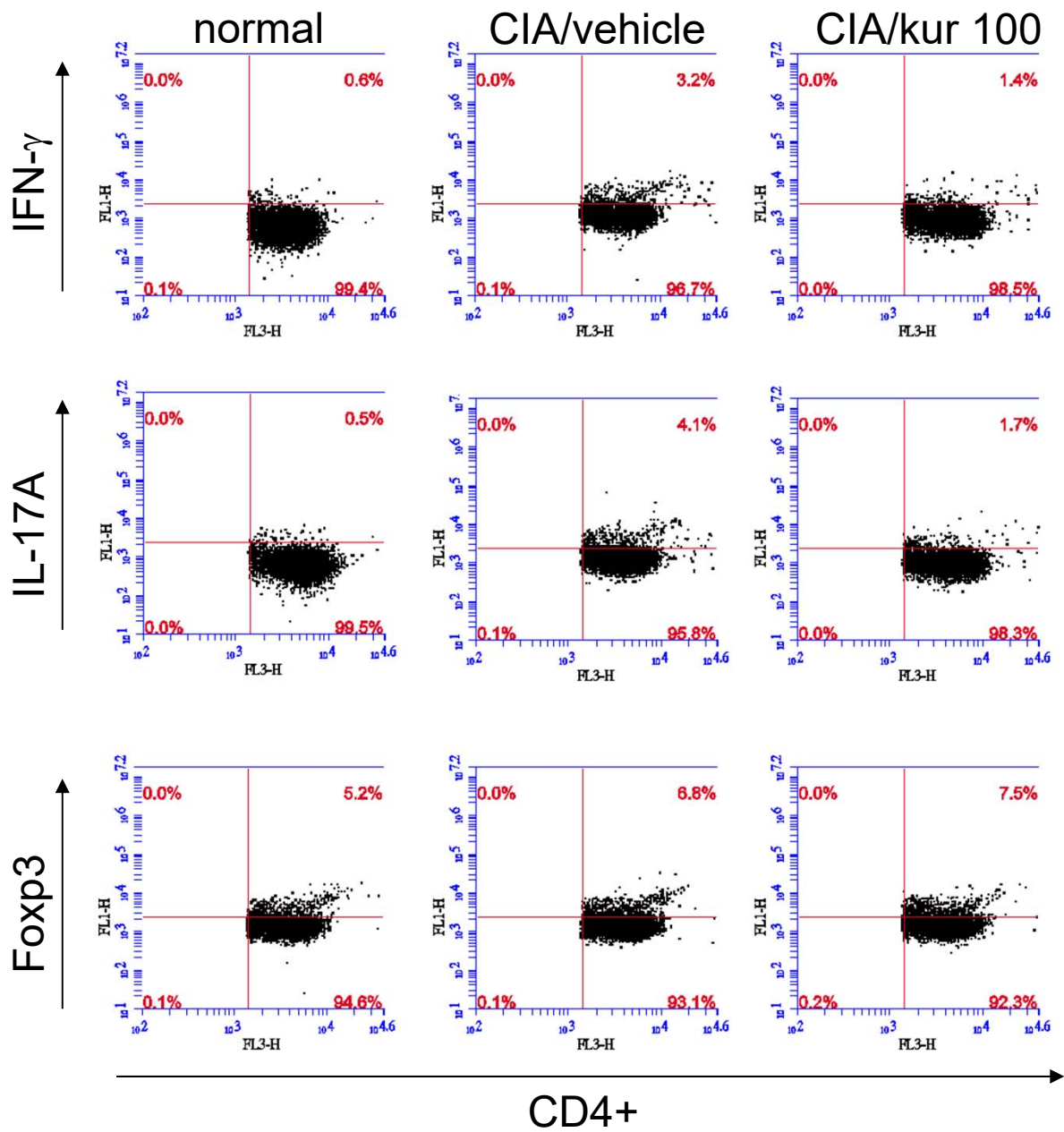

Supplemental Fig S2

A

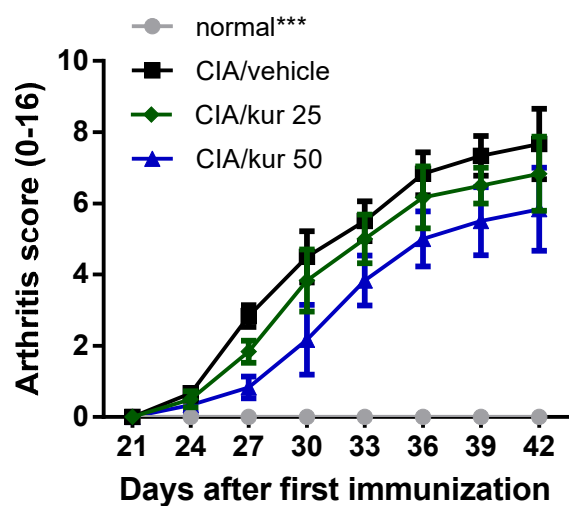

B

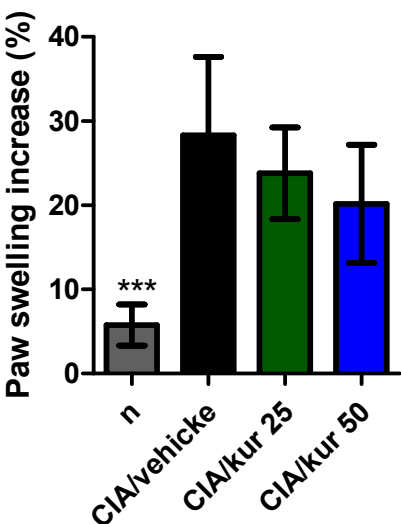

Supplement: Supplementary file 1 [file ijms-22-04002-s001.pdf]
